# 672. Impact of Multistep Testing Algorithm on Hospital Acquired *Clostridioides difficil*e Infection Rates

**DOI:** 10.1093/ofid/ofad500.734

**Published:** 2023-11-27

**Authors:** Adriana C Betancourth, Arshpal Gill, Salman Bangash, Charmaine Abalos, Nitin Bhanot, Zaw Min, Matthew A Moffa, Nathan R Shively, Thomas L Walsh

**Affiliations:** Allegheny Health Network, Pittsburgh, Pennsylvania; Allegheny Health Network, Pittsburgh, Pennsylvania; Allegheny General Hospital, Pittsburh, Pennsylvania; Allegheny Health Network, Pittsburgh, Pennsylvania; Allegheny Health Network, Pittsburgh, Pennsylvania; Allegheny General Hospital, Pittsburh, Pennsylvania; Allegheny Health Network, Pittsburgh, Pennsylvania; Allegheny Health Network, Pittsburgh, Pennsylvania; Allegheny Health Network, Pittsburgh, Pennsylvania

## Abstract

**Background:**

*Clostridioides difficile* infection (CDI) is one of the leading causes of hospital-acquired (HA) infections. Clinically distinguishing true CDI versus colonization is challenging. Our center previously used a polymerase chain reaction (PCR) assay as a standalone test for diagnosing CDI. While PCR is very sensitive for diagnosing CDI, it cannot distinguish between CDI and asymptomatic colonization. A multistep testing algorithm, which consists of an initial *C. difficile* PCR test followed by a reflex enzyme immunoassay (EIA) for glutamate dehydrogenase (GDH) and Toxin A/B testing, was implemented in our institution. We conducted this study to determine the rates of HA-CDI and *C. difficile* colonization based on laboratory results.

**Methods:**

This retrospective observational study was conducted at Allegheny General Hospital (Pittsburgh, PA, US). Patients with a positive *C. difficile* PCR assay at day 3 or later from admission date were included for analysis. The pre-intervention period was from August 2021 to February 2022 and post-intervention period was from August 2022 to February 2023. Before the intervention, results for *C. difficile* testing were reported as PCR positive (PCR+) or negative (PCR-). On July 2022, a multistep testing algorithm was implemented with an initial *C. difficile* PCR assay; if results were positive, a reflex EIA for GDH and Toxin A/B testing was performed and resulted as follows: Figure 1.

The HA-CDI rates before and after implementation of a multistep testing algorithm were compared.

**Figure:**
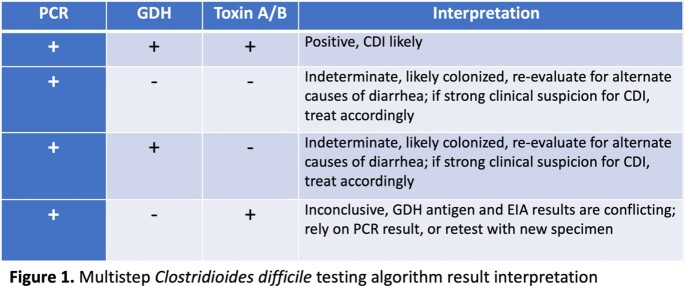

**Results:**

A total of 112 unique patients were included. HA-CDI in the post intervention period decreased by 53% after the intervention (N=57 [pre-intervention] versus N=26 [post-intervention]; P < 0.001). During the post-intervention period, there were 55 PCR+ *C. difficile* assays, of which 29 (53%) were resulted as "indeterminate, likely *C. difficile* colonization." Of the 29 indeterminate results, 23 (79%) still received CDI therapy.

**Figure:**
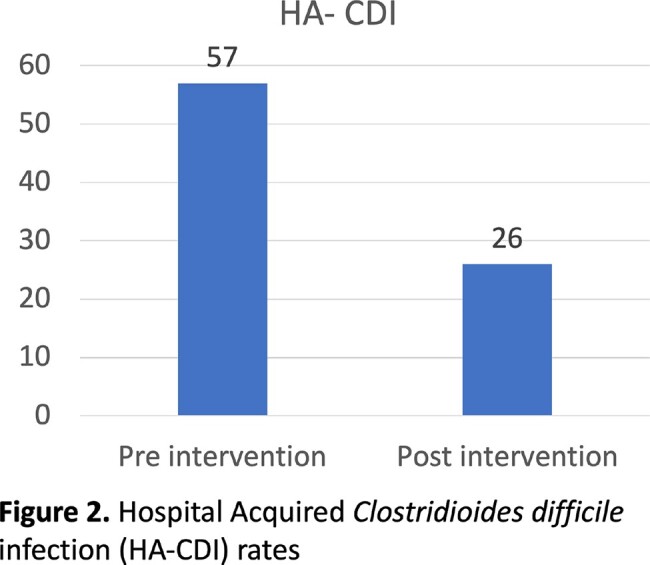

**Conclusion:**

Our study demonstrated that a multistep *C. difficile* testing algorithm significantly reduced the rates of HA-CDI in our institution. We found that the majority of patients still received CDI therapy despite having a negative EIA toxin result. We plan to analyze the clinical impact of this multistep testing algorithm in a follow-up study.

**Disclosures:**

**Thomas L. Walsh, MD**, Accelerate Diagnostics: Advisor/Consultant

